# Mechanism of Abnormal Chondrocyte Proliferation Induced by Piezo1-siRNA Exposed to Mechanical Stretch

**DOI:** 10.1155/2020/8538463

**Published:** 2020-11-04

**Authors:** Yi Sun, Ping Leng, Dawei Li, Huanshen Gao, Zhenghui Li, Chenkai Li, Haining Zhang

**Affiliations:** ^1^Department of Joint Surgery, The Affiliated Hospital of Qingdao University, Qingdao 266000, China; ^2^Department of Pharmacy, The Affiliated Hospital of Qingdao University, Qingdao 266000, China

## Abstract

**Objective:**

To investigate the effect of small interfering RNA targeting mechanosensitive ion channel protein Piezo1 (Piezo1-siRNA) on abnormal chondrocyte proliferation exposed to mechanical stretch.

**Methods:**

Construct and screen effective Piezo1-siRNA sequences and explore an appropriate method to transfect lentiviral vector into chondrocytes exposed to mechanical stretch. Western blot and RT-PCR were used to detect the mRNA and protein expression of Piezo1, Kif18A, and *β*-tubulin, respectively. Flow cytometry was used to measure the changes in the chondrocyte cycle. The proliferation of chondrocyte was evaluated by cell counting kit-8.

**Results:**

According to the mRNA and protein expression of Piezo1, the effective siRNA sequence was successfully screened. Compared with the 0 h group, mechanical stretch upregulated the expression of Piezo1, Kif18A, and *β*-tubulin, resulting in chondrocyte cycle arrest and eventually inhibiting chondrocyte proliferation. Moreover, Piezo1-siRNA transfection effectively blocks this process and promotes the proliferation of chondrocyte.

**Conclusion:**

Piezo1-siRNA can reduce the inhibition of chondrocyte proliferation induced by mechanical stretch via downregulating the expression of Kif18A and inhibiting the depolymerization of microtubules. Piezo1-siRNA plays a protective role in chondrocytes, which provides a potential method for the treatment of OA under abnormal mechanical stimulation.

## 1. Introduction

Osteoarthritis (OA) is a degenerative disease of bone and articular cartilage, which is caused by mechanical and biological factors [1–3]. Degenerative changes of articular cartilage are closely related to abnormal proliferation of chondrocyte [4, 5], in which mechanical stretch stress plays a crucial role [6]. It has been reported that the proliferation of chondrocytes under different mechanical stretch was varied, and the proliferation of chondrocytes decreased with the extension of mechanical stretch time. However, the potential molecular mechanisms of abnormal proliferation of chondrocyte exposed to mechanical stretch have not been elucidated [7].

Piezo1 is a novel type of nonselective mechanically sensitive ion channel which is different from other ion channels in structure and gating mechanism [8, 9]. It converts mechanical signals into chemical signals and engages in cell proliferation, apoptosis, and metabolism [10]. Previous studies have reported that the expression of Piezo1 in chondrocyte exposed to different mechanical stretch is various, and chondrocyte proliferation is closely associated with the time of mechanical stretch [11]. However, whether Piezo1 is involved in mechanical stretch-induced abnormal proliferation of chondrocytes is still unclear.

Microtubule kinesin, a kind of motor protein with ATPase activity, plays a significant role in the dynamic changes of microtubules and intracellular material transport [12]. Recently, it has been found that Kif18A, a member of the kinesin-8 family, participates in many biological functions of cells, such as spindle formation, chromosome separation, cell division, microtubule polymerization, and depolymerization [13]. The morphology and microtubule of chondrocytes were changed under mechanical stretch [14]. Combined with previous studies, we propose a hypothesis that mechanical stretch can upregulate the expression of Piezo1 in chondrocytes and then activate the microtubule kinesin Kif18A, resulting in increased microtubule depolymerization and cell proliferation inhibition. To confirm the hypothesis, we conducted an experiment to investigate the expression of Piezo1, Kif18A, and *β*-tubulin in chondrocytes exposed to mechanical stretch. The cell cycle and proliferation of chondrocyte were also measured by flow cytometry and cell counting kit-8. In addition, siRNA was used to silence the expression of Piezo1 in order to provide a potential drug target for chondrocyte protection under mechanical stretch.

## 2. Materials and Methods

### 2.1. Chondrocyte Culture

This project was approved by the Ethics Committee of the Affiliated Hospital of Qingdao University. All patients were informed and signed consent. Chondrocytes were harvested from articular cartilage derived from the OA patients undergoing total knee arthroplasty. Cartilage tissue was briefly washed with phosphate-buffered saline (PBS) containing 400 U/ml penicillin and 0.4 mg/ml streptomycin (Hyclone, USA) for 3 times. The samples were cut into 1 mm^3^ granules under aseptic conditions. After trypsin and collagenase (Solaibio, China) sequential digestion for 4 h, the cells were collected after 1000 r/min centrifugation. The cells were added to DMEM (Gibco, USA) containing 15% fetal bovine serum (Gibco, USA). After counting, the cells were inoculated in a 25 cm^2^ culture bottle and cultured in a 5% CO_2_ incubator (Sanyo, Japan) at 37°C in a humid atmosphere.

### 2.2. Chondrocyte Identification

The primary chondrocytes were inoculated into the 12-well plate containing glass cover slips. When the cell fusion rate reached 70, the chondrocytes were fixed with 4% paraformaldehyde (Solaibio, China) for 15 min, followed by permeabilization with 0.2% Triton X-100 (MP Biomedicals, USA) for 5 min. After washing, the cells were preincubated for 30 min in 5% BSA to prevent nonspecific antibody binding. The human type II collagen (Novus Biologicals, USA) antibody was incubated overnight at 4°C. AlexaFluor 488 Goat Anti-Rabbit lgG (diluted 1 : 2000; Invitrogen, USA) was added to incubate for 30 min. The dye solution of diaminobenzidine (DAB) was dyed and observed under an inverted optical microscope (Olympus GX51, Olympus, Japan).

The cells were fixed with 4% polyformaldehyde for 1 h, then washed by water for 15 min and by distilled water once, and then stained with 1% toluidine blue for 3 h. 95% alcohol was added, the excess dye was washed out, and the cells were observed under an inverted optical microscope.

### 2.3. Construction of Piezo1-siRNA

The construction of the virus vector was completed by Gemma gene (Shanghai). All sequences were specific oligonucleotide chains with 63 bD (Table 1). The vector pHBLV-U6-ZsGreen-PGK-Puro cut the negative control sequence away to obtain the skeleton by BamHI, EcoRI enzyme, and then, the target sequence was inserted into the U6 promoter to regulate its expression. The lentivirus vector contained ZsGreen (GFP) regulated by CMVIE promoter and puro resistant gene initiated by PKG promoter. Virus titer was determined by a hole-by-hole dilution titer assay (1.0 × 10^8^ TU/ml) after virus collection.

### 2.4. Screening of Lentivirus Vectors

The chondrocytes were divided into 5 groups: a (blank control), b (Piezo1-siRNA1), c (Piezo1-siRNA2), d (Piezo1-siRNA3), and e (negative control). Totally, 50 *μ*l lentivirus vector, containing each interference sequence and negative control sequence, was added to the culture medium, respectively. The titer is 1 × 10^8^ TU/ml. The same amount of culture medium solution was added in group a. After 48 h, the cells were observed and counted under a fluorescence microscope. After 96 h, the cells were collected. Western blot and RT-PCR were used to detect the mRNA and protein expression of Piezo1, respectively.

### 2.5. Construction of Mechanical Stretch Model of Chondrocytes

The chondrocytes were inoculated into the Flexcell aseptic membranous 6-well plate (Flexcell, USA), cultured in 2.5 ml low-glucose DMEM containing 10% fetal bovine serum each well. The chondrocytes were divided into three groups: blank control group, lentivirus empty vector group, and lentivirus interference sequence group. When cell fusion reaches 40%, the selected effective lentivirus was added and the empty vector virus 50 *μ*l per well. The virus titer is 1 × 10^8^ TU/ml. The culture plates were placed in the multichannel cell stretch stress loading system FX-4000T (Flexcell, USA) in a 5% CO_2_ incubator at 37°C in a humid atmosphere. Because of the different stretch times applied to cells, each group was divided into five subgroups, and the cyclic stretch of 0 h/2 h/12 h/24 h/48 h was loaded, respectively, with the amplitude of 20% and the period of 10 times/min.

### 2.6. Detection of Cell Proliferation by Cell Counting Kit-8 (CCK-8)

The cells of each group were obtained by trypsin digestion and centrifugation. After counting, 5 *μ*l cell suspensions were inoculated into a 96-well plate and supplemented with culture medium. After 6 h of culture, 10 *μ*l CCK-8 solution was added to each well and the number of cells was measured. The operation was carried out according to the manufacturer's instructions.

### 2.7. Detection of Cell Cycle by Propidine Iodide (PI) Single Staining Flow Cytometry

Each group of cells was washed twice with 1 ml PBS. The cells were immobilized by absolute ethyl alcohol at 4°C for 2 h. Each sample was resuspended with 200 *μ*l precooled PBS, then added with 10 *μ*l RNA enzyme (Beyotime, China) and 25 *μ*l PI staining solution (Beyotime, China). The cells were fully suspended at 37°C for 30 min. Red fluorescence and light scattering were detected by flow cytometry at 488 nm wavelength. The cell DNA content and light scattering analysis were analyzed by FlowJo v10 software, and the percentage of cells in each cycle was analyzed by a self-cell cycle model.

### 2.8. RT-PCR

Each group was added RNAiso (TaKaRa, Japan) to extract the total RNA. The PrimeScript RT reagent kit (TaKaRa, Japan) was used to reverse RNA to cDNA. Then, the SYBR Premix Ex Taq II kit (TaKaRa, Japan) was used for RT-PCR. The CT values of Piezo1, Kif18A, *β*-tubulin, and GAPDH genes were obtained by the FTC-2000 system (Applied Biosystems, China), and the parameters were as follows: predenaturation: 95°C for 30 seconds, 1 cycle; PCR: 95°C for 5 seconds, 60°C for 30 seconds, and 72°C for 30 seconds for 40 cycles. The relative expression of the target gene was calculated by 2^−*ΔΔ*Ct^. GAPDH, Piezo1, Kif18A, and *β*-tubulin primers were produced by Shanghai Bioengineering Company (Table 2).

### 2.9. Western Blot

Cells were lysed with the precooled RIPA lysate (Solarbio, China). The supernatant was obtained after centrifugation at 4°C for 12000 r/min. After adding an appropriate amount of sample buffer to the protein sample, the protein samples and standard protein marker were added to polyacrylamide gel and then electrophorized. After electrophoresis, the protein was transferred to the polyvinylidene fluoride membrane. The membranes immersed in the target protein antibody (dilution concentration 1 : 5000; Abcam, China) were incubated overnight at 4°C. The second antibody (dilution concentration 1 : 10000; Abcam, China) was added for 1 h. The enhanced chemiluminescence reagent was used to detect the target proteins. GAPDH was used as the internal control.

### 2.10. Statistical Analysis

The data were processed by SPSS19.0 statistical software, the measurement data were expressed as the mean ± standard deviation (SD), a *t-*test was used when the two independent groups of measurement data were compared, and the percentage of the cell cycle was tested by the Poisson distribution data *z-*test. One-way ANOVA was used to compare the mean values of multiple samples. *P* < 0.05 was defined as statistically significant.

## 3. Results

### 3.1. Cell Identification

**Figure 1:**
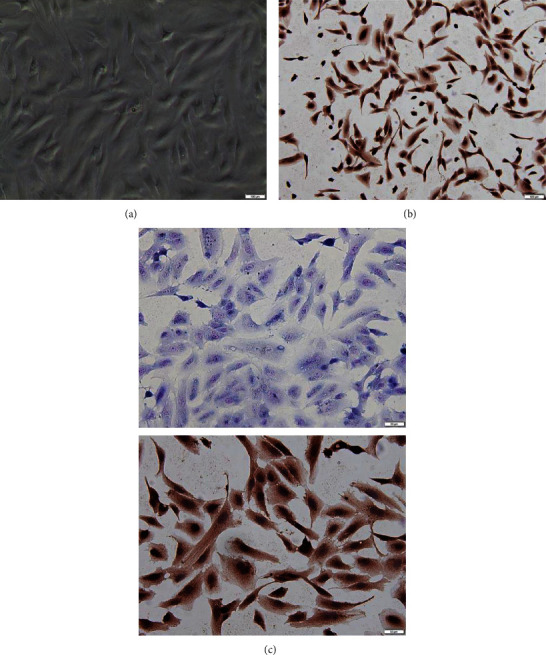
Cell culture and identification: (a) second-generation chondrocytes, mononuclear, and polynucleolus; (b) type II collagen immunohistochemical staining; (c) toluidine blue staining of chondrocytes.

The chondrocytes were fusiform or polygonal, mononuclear, or polynucleolar and adherent to the wall (Figure 1(a)). Immunohistochemical staining showed that the cytoplasm of chondrocytes was brown and the nucleus was blue, indicating that type II collagen exists in the cytoplasm of chondrocytes (Figure 1(b)). Toluidine blue staining showed that the cytoplasm of chondrocytes was purple blue and the background was light blue (Figure 1(c)) indicating that glycosaminoglycan exists in the matrix surrounding the chondrocytes.

### 3.2. Lentivirus Transfection into Chondrocytes and Screening of Effective Sequences

**Figure 2:**
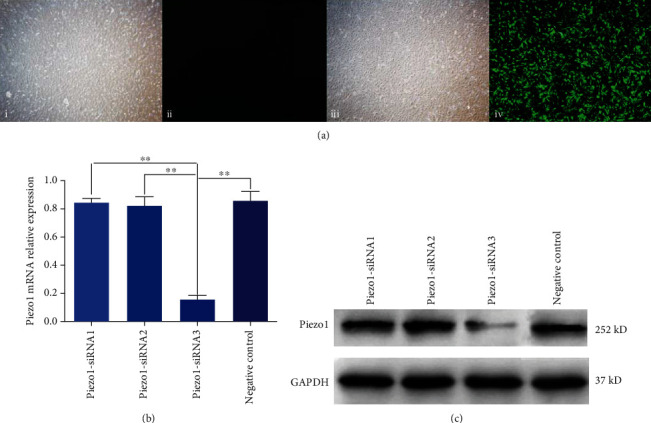
Screening of effective lentivirus sequences. (a) The efficiency of lentivirus transfection in each group, green fluorescence as lentivirus vector; i and ii show a group of lentivirus-free vectors observing cells in the same field of vision under normal and fluorescent light sources; iii and iv indicated that the lentivirus vector group was added to observe the same visual field cells under normal and fluorescent light sources, and the lentivirus was transferred into the cells. (b) The relative mRNA expression of Piezo1. (c) The results of protein expression of Piezo1 protein.

After 72 h of transfection, the complex number of infection was calculated and the transfection efficiency was measured. It was calculated that the average number of infection was 30 ± 4 TU/cell in each group with 50 *μ*l viral fluid, and the transfection efficiency was 0%, 91.88%, 92.33%, and 94.23%, respectively (Figure 2(a)). The mRNA expression of Piezo1 in the Piezo1-siRNA3 group was significantly decreased than that in the negative group (*P* < 0.01) (Figure 2(b)). Similar results were also observed in the protein expression of Piezo1 (Figure 2(c)), so the Piezo1-siRNA3 could be used as the effective sequence of the Piezo1 silencing vector.

### 3.3. Detection of Cell Proliferation by CCK-8

**Figure 3:**
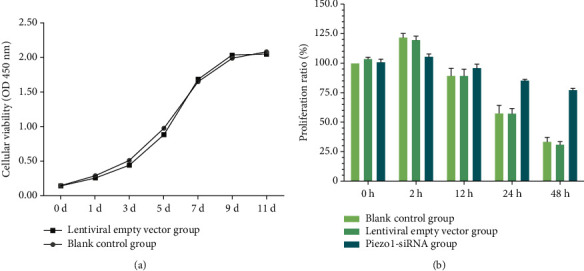
Detection of cell proliferation by cell counting kit-8. (a) Detecting the effect of lentivirus on cell proliferation under different days, there was no significant difference in the number of living cells between the two groups. (b) The results of cell proliferation in each group under different stretch.

The OD values of the blank control group and the lentivirus empty vector group were measured at different time points to make the broken line diagram (Figure 3(a)). According to the graph, there was no difference between the OD values of the two groups at each time point (*P* > 0.05). After the lentiviral interference vector Piezo1-siRNA3 was added, the cell proliferation showed different changes in different mechanical stress groups. With the extension of stretching time, cell proliferation first increased and then decreased. The cell proliferation was the highest at 2 h and the lowest at 48 h. However, after mechanical stretch for 2 h, the proliferation rate of the Piezo1-siRNA group was lower than that in the blank control group after 2 h, and there was a significant difference between the Piezo1-siRNA group and the blank control group (*P* < 0.05) (Figure 3(b)).

### 3.4. Detection of Cell Cycle by Flow Cytometry

**Figure 4:**
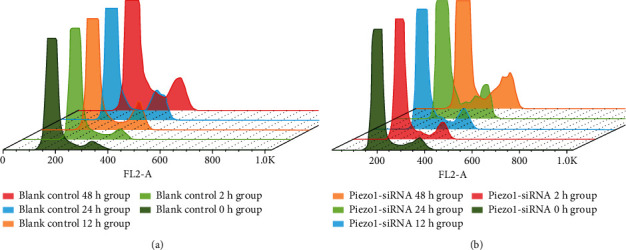
The cell cycle composite columnar map analyzed by FlowJo v10; the high peak represented the G0/G1 phase, and the low peak represented the G2/M phase. The values obtained are shown in Table 3. (a) The periodic distribution of different stretch times in the blank control group. (b) The periodic distribution of different stretch times in the Piezo1-siRNA group.

Cell cycle results of the blank control group and Piezo1-siRNA group are shown in Figure 4 and Table 3. In the control group, there was no difference in the percentage of cells in the G2/M phase (*P* > 0.05). Compared with the 0 h blank control group, the percentage of G2/M phase cells in the 12 h/24 h/48 h blank control group was significantly different (*P* < 0.05), and the percentage of the G2/M phase cell in the 24 h blank control group was the highest, which was significantly higher than that in the 0 h blank control group. There was no difference in the percentage of G2/M phase cells in the Piezo1-siRNA group under different stretch times (compared with the 0 h group, *P* > 0.05).

### 3.5. RT-PCR Results of Piezo1, Kif18A, and *β*-Tubulin

**Figure 5:**
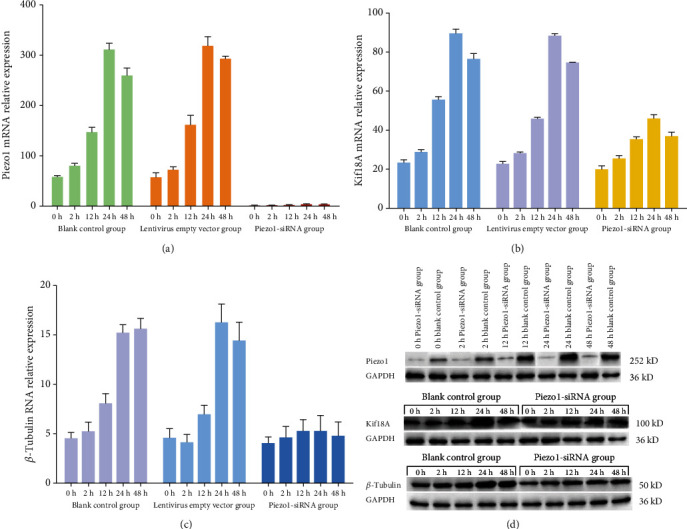
The expression of Piezo1, Kif18A, and *β*-tubulin: (a) the mRNA expression of Piezo1; (b) the mRNA expression of Kif18A; (c) the mRNA expression of *β*-tubulin; (d) the protein expression of Piezo1, Kif18A, and *β*-tubulin.

The mRNA expression characteristics of Piezo1 and Kif18A in the blank control group and empty vector group were basically the same, showing the first increase and then decrease, and reached the maximum expression after 24 h mechanical stretch (Figures 5(a) and 5(b)). Upon transfection with Piezo1-siRNA, there was no significant difference in mRNA expression of Piezo1 among the groups (*P* > 0.05), and the expression of Kif18A and *β*-tubulin decreased (*P* > 0.05, Figure 5(c)).

### 3.6. Western Blotting Results of Piezo1, Kif18A, and *β*-Tubulin

The expression of Piezo1 in chondrocytes changed with the extension of mechanical stretch time, and the expression of Piezo1 protein was significantly inhibited by transfection of siRNA. The protein expression of Kif18A and *β*-tubulin in chondrocytes exposed to mechanical stretch was consistent with that of mRNA expression. After transfection with Piezo1-siRNA, the expression of Kif18A and *β*-tubulin decreased significantly (*P* < 0.05). (Figure 5(d)).

## 4. Discussion

Previous studies have reported that Piezo1 can mediate chondrocyte apoptosis by regulating the polymerization and depolymerization of cytoskeleton. However, it is not clear whether Piezo1 plays a role in the proliferation of chondrocytes exposed to mechanical stretch [15]. Therefore, the purpose of this study is to investigate the biomechanical effects of chondrocytes cultured in vitro, focusing on whether Piezo1 regulates the proliferation of chondrocytes, and the potential role of cytoskeleton and kinesin in this process. In vitro exposure to mechanical stretch of chondrocyte proliferation results showed that moderate stretch (2 h) promoted the proliferation of chondrocytes, but excessive stretch led to abnormal proliferation of chondrocytes. Transfection of Piezo1-siRNA effectively reduced the abnormal proliferation of chondrocytes caused by excessive mechanical stretch. In addition, we found that mechanical stretch promoted the expression of Piezo1 to a certain extent, which made the inhibitory effect of mechanical stretch on cell proliferation more significant. These results confirm that Piezo1 plays an important role in regulating the proliferation of chondrocytes under mechanical stimulation. Inhibition of Piezo1 effectively reduced the injury of chondrocytes induced by mechanical stretch.

Kif18A regulates the stability of microtubules through the activity of microtubule depolymerization enzyme, resulting in cytoskeleton changes [16]. Kif18A expression was significantly increased in the mitotic phase of eukaryotic cells, indicating that it may participate in the process of mitosis, but the specific mechanism is unclear [17, 18]. In recent years, some researchers have found that Piezo1 is closely associated with cytoskeleton. Gottlieb et al. [19] found that the current intensity of the cell membrane decreased by stimulating the channel on the cell surface pretreated with cytochalasin D, indicating that actin is responsible for the transmission of mechanical stimulation. Previous studies have reported that cytoskeleton plays a mechanical protective role on Piezo1, which means that intact cytoskeleton increases the difficulty of activating Piezo1 [20, 21]. The destruction of cytoskeleton integrity makes the same mechanical stretch that mediates larger cell morphological changes, which enhances the activation of Piezo1 [22]. Combined with the research reports [13], we speculate that the abnormal proliferation of chondrocytes under mechanical stretch may be closely related to cytoskeleton and kinesin. Our results showed that the protein and mRNA expression of Kif18A increased with the extension of mechanical stretch time. Compared with the empty vector group, the expression of Kif18A in the Piezo1-siRNA group was markedly decreased, which implied that the activation of Piezo1 could promote the overexpression of Kif18A. The activation mechanism may be related to the changes in the intracellular calcium level after the opening of channels, which still needs to be explored. We also confirmed that the expression of *β*-tubulin upregulated with the extension of mechanical stretch time, and the expression of *β*-tubulin in the empty vector group was higher than that in the Piezo1-siRNA group. We speculate that activated Kif18A destroys the integrity of cytoskeleton by playing the role of microtubule depolymerizing enzyme, thus promoting the expression of Piezo1 and forming a positive feedback loop [23]. The expression of Piezo1 in the 24 h group was significantly higher than that in the 12 h group, which supported our hypothesis. Interestingly, compared with the mechanical stretch group for 24 h, the Piezo1 expression and cell viability of the 48 h mechanical stretch group were lower. Combined with the experimental results of Grishchuk [16], we believe that excessive mechanical stretch may exceed the tolerance of cells, causing cell proliferation arrest and inducing apoptosis. Cell cycle analysis showed that the percentage of G2/M phase cells in the 24 h mechanical stretch group was significantly higher than that in the 0 h group, suggesting that Kif18A expression increases and cytoskeleton depolymerization promotes cell cycle arrest, which is consistent with the results of Huang et al. [24] in the study of Kif18s-induced microtubule depolymerization leading to chromosome arrangement defects and mitotic stagnation after excessive aggregation of microtubules at the positive tip of microtubules. It is concluded that Piezo1 can induce cytoskeleton destruction and inhibit the proliferation of chondrocytes by activating Kif18A to depolymerize microtubules.

## 5. Conclusions

In this study, we found that Piezo1-siRNA effectively interferes with Piezo1 expression and alleviates mechanical stretch-mediated inhibition of chondrocyte proliferation. Our conclusion is that mechanical stretch activates Piezo1, resulting in the overexpression of Kif8a, which leads to microtubule depolymerization, destroys the integrity of cytoskeleton, and inhibits the mitosis of cells. Transfection of Piezo1-siRNA effectively blocked the pathway and corrected the abnormal proliferation of cells. Therefore, Piezo1-siRNA may protect the cartilage of patients with osteoarthritis, which provides a potential method for the treatment of persistent progression of OA under abnormal mechanical stimulation. However, the changes in intracellular chemical signals, the response of cytoskeleton to Piezo1, and whether there are other ways to inhibit cell proliferation after Piezo1 activation need further study and discussion.

## Figures and Tables

**Table 1:** Interfering sequences and negative control sequences.

Oligo name	Oligo sequence
Piezo1-siRNA1	5′-GATCCGCGTCATCATCGTGTGTAAGATTCAAGAGATCTTACACACGATGATGACGCTTTTTTG-3′ 3′-AATTCAAAAAAGCGTCATCATCGTGTGTAAGATCTCTTGAATCTTACACACGATGATGACGCG-5′
Piezo1-siRNA2	5′-GATCCGCGTCTTCCTTAGCCATTACTTTCAAGAGAAGTAATGGCTAAGGAAGACGCTTTTTTG-3′ 3′-AATTCAAAAAAGCGTCTTCCTTAGCCATTACTTCTCTTGAAAGTAATGGCTAAGGAAGACGCG-5′
Piezo1-siRNA3	5′-GATCCGCCTCAAGTACTTCATCAACTTTCAAGAGAAGTTGATGAAGTACTTGAGGCTTTTTTG-3′ 3′-AATTCAAAAAAGCCTCAAGTACTTCATCAACTTCTCTTGAAAGTTGATGAAGTACTTGAGGCG-5′
Negative control	5′-GATAGCGTCCATCATCGTGTGTAAGATTCAAGAGATCTTACACACGATGATGACGCTTTTTTG-3′ 3′-AATAGCGTCAAGCGTCATCATCGTGTGTAAGATCTCTTGAATCTTACACACGATGATGACGCG-5′

**Table 2:** Oligo sequences of the target genes.

Oligo name		Oligo sequence
Piezo1	Forward primer	5′-CATCTTGGTGGTCTCCTCTGTCT-3′
	Reverse primer	5′-CTGGCATCCACATCCCTCTCATC-3′
Kif18A	Forward primer	5′-GCAGCTCAATGATTCTCTTAGC-3′
	Reverse primer	5′-TTACACTTCGAGCTCTTGATGT-3′
*β*-Tubulin	Forward primer	5′-ATTCCAACCTTCCAGCCTGC-3′
	Reverse primer	5′-CCAGAACTTGGCACCGATCT-3′
GAPDH	Forward primer	5′-GCACCGTCAAGGCTGAGAAC-3′
	Reverse primer	5′-TGGTGAAGACGCCAGTGGA-3′

**Table 3:** Percentage of cells in the blank control group and the lentiviral interference group at different stress times.

Group name		< diploid (%)	Cell cycle (%)			> tetraploid (%)
			G0/G1	S	G2/M	
Blank control	48 h group	7.89	61.90	20.80	^∗∗^6.37	0.48
Blank control	24 h group	4.17	71.70	13.30	^∗∗^8.16	1.05
Blank control	12 h group	10.80	62.60	17.10	^∗∗^5.65	0.54
Blank control	2 h group	10.90	68.70	11.50	5.31	0.19
Blank control	0 h group	16.60	64.60	27.50	4.06	2.01
Piezo1-siRNA	48 h group	24.70	51.90	14.90	^∗^4.86	0.33
Piezo1-siRNA	24 h group	2.34	70.20	20.10	^∗^4.85	0.18
Piezo1-siRNA	12 h group	6.65	78.20	8.96	^∗^4.00	0.38
Piezo1-siRNA	2 h group	4.19	72.10	15.80	^∗^5.68	0.32
Piezo1-siRNA	0 h group	8.22	58.10	25.00	4.61	0.83

^∗∗^vs. blank control 0 h group, *z-*test, *P* < 0.05; ^∗^vs. Piezo1-siRNA 0 h group, *z*-test, *P* > 0.05.

## Data Availability

The data used to support the findings of this study are available from the corresponding author upon request.
